# Phytochemical component and toxicological evaluation of purple sweet potato leaf extract in male Sprague–Dawley rats

**DOI:** 10.3389/fphar.2023.1132087

**Published:** 2023-04-03

**Authors:** Ahmad Safiyyu’d-din Bin Hisamuddin, Ruth Naomi, Khairul Aiman Bin Manan, Hasnah Bahari, Muhammad Dain Yazid, Fezah Othman, Hashim Embong, Siti Hadizah Jumidil, Mohd Khairi Hussain, Zainul Amiruddin Zakaria

**Affiliations:** ^1^ Borneo Research on Algesia, Inflammation and Neurodegeneration (BRAIN) Group, Faculty of Medicine and Health Sciences, Universiti Malaysia Sabah, Kota Kinabalu, Sabah, Malaysia; ^2^ Department of Biomedical Sciences, Faculty of Medicine and Health Sciences, Universiti Putra Malaysia, Serdang, Malaysia; ^3^ Department of Human Anatomy, Faculty of Medicine and Health Sciences, Universiti Putra Malaysia, Serdang, Malaysia; ^4^ Centre for Tissue Engineering and Regenerative Medicine, Faculty of Medicine, Universiti Kebangsaan Malaysia, Kuala Lumpur, Malaysia; ^5^ Department of Emergency Medicine, Faculty of Medicine, Universiti Kebangsaan Malaysia, Kuala Lumpur, Malaysia

**Keywords:** medicinal plant, purple sweet potato, plant extract, phytochemicals, bioactive compounds, toxicity, animal study

## Abstract

This study assessed the toxicity of lutein-rich purple sweet potato leaf (PSPL) extract in male Sprague–Dawley rats.

**Methods and study design:** A total of 54 adult male Sprague–Dawley rats were used. For the acute toxicity study, three rats in the acute control group were fed 2,000 mg/kg of PSPL for 14 days. The subacute toxicity study included six rats each in four groups administered 50, 250, 500, or 1,000 mg/kg for 28 days and observed for further 14 days without treatment in the subacute control and subacute satellite groups. Changes in body weight; blood biochemistry; hematological parameters; relative organ weight; and histological sections of the heart, kidney, liver, pancreas, aorta, and retina were observed for signs of toxicity.

**Results:** The gradual increase in weekly body weight, normal level full blood count, normal liver and kidney profile, relative organ weight, and histological sections of all stained organ tissue in the treated group compared with the acute, subacute, and satellite control groups demonstrated the absence of signs of toxicity.

**Conclusion:** Lutein-rich PSPL extract shows no signs of toxicity up to 2,000 mg/kg/day.

## 1 Introduction

Functional foods have attracted research attention due to safety concerns. Any natural product containing bioactive molecules integrated into food is known as a functional food. In this context, one of the widely used functional food currently being studied is PSPL (Khairani et al., 2022). PSPL, also known as *Ipomoea batatas* (L.) Lam. is a dicotyledonous plant that belongs to the botanical family of Convolvulaceae. It is widely known for its natural medicinal properties, with the leaf itself containing up to 130 known metabolites (Naomi et al., 2021). This plant usually grows well in tropical and subtropical regions and is widely cultivated in more than 100 countries worldwide (Alam, 2021). In China alone, PSPL cultivation exceeds more than 76% of the world’s production (Nguyen et al., 2021). This is attributed due to the plant’s high drought tolerance and ability to grow in changing climates (Shekhar et al., 2014). Purple sweet potato is the sixth most common crop worldwide, with the highest production in the southeastern parts of India (Alam, 2021). Approximately, 115 million metric tons of sweet potatoes are cultivated annually through vegetative propagation (Mohanraj and Sivasankar, 2014). Most parts of the purple sweet potato can be eaten since they contain nutritional and bioactive compounds. Purple sweet potatoes are significantly rich in lutein, proteins, vitamins, minerals, fibers, and β-carotene compared to other commonly available major greens in Asian countries (Alam, 2021). A recent study showed that PSPL is a strong antioxidant with radical scavenging ability due to the presence of natural bioactive compounds such as anthocyanin (Yuzhi Jiao Studies on antioxidant, 2012). Due to its natural essences, PSPL has been formulated into a probiotic to make its consumption easier and so that more people can experience the maximum benefits (Kim et al., 2015). Some of the other identified minerals in PSPL include sodium, potassium, calcium, magnesium, iron, copper, manganese, and zinc (Sun et al., 2014).

The most well-known health benefit of purple sweet potato is its anti-diabetic effects due to xenobiotic phytochemical constituents (Strugała et al., 2019). Some of the plant’s genotypes are associated with nutraceutical value (Escobar-Puentes et al., 2022). Despite its known functional value, the plant is underutilized, possibly due to safety concerns regarding the consumption of the natural plant and the lack of scientific data on its therapeutic use. In some countries, *I. batatas* is completely avoided by women due to concerns about unintentional miscarriages. However, no specific study has reported that *I. batatas* is toxic for consumption or its adverse effects in pregnant subjects (Gunn et al., 2013). A previous study reported that purple sweet potato contains oxalic acid, which has the potential be converted into oxalate stones in the urinary tract. Thus, to avoid unwanted adverse effects, individuals with a history of kidney stones should avoid eating purple sweet potatoes (Noonan and Savage, 1999). However, this remains unconfirmed since no recent data have been reported regarding the safety and efficacy of purple sweet potatoes. Hence, the present study evaluated the toxicological effects of PSPL extract in male Sprague–Dawley rats.

## 2 Results

### 2.1 Lutein quantification

**FIGURE 1:**
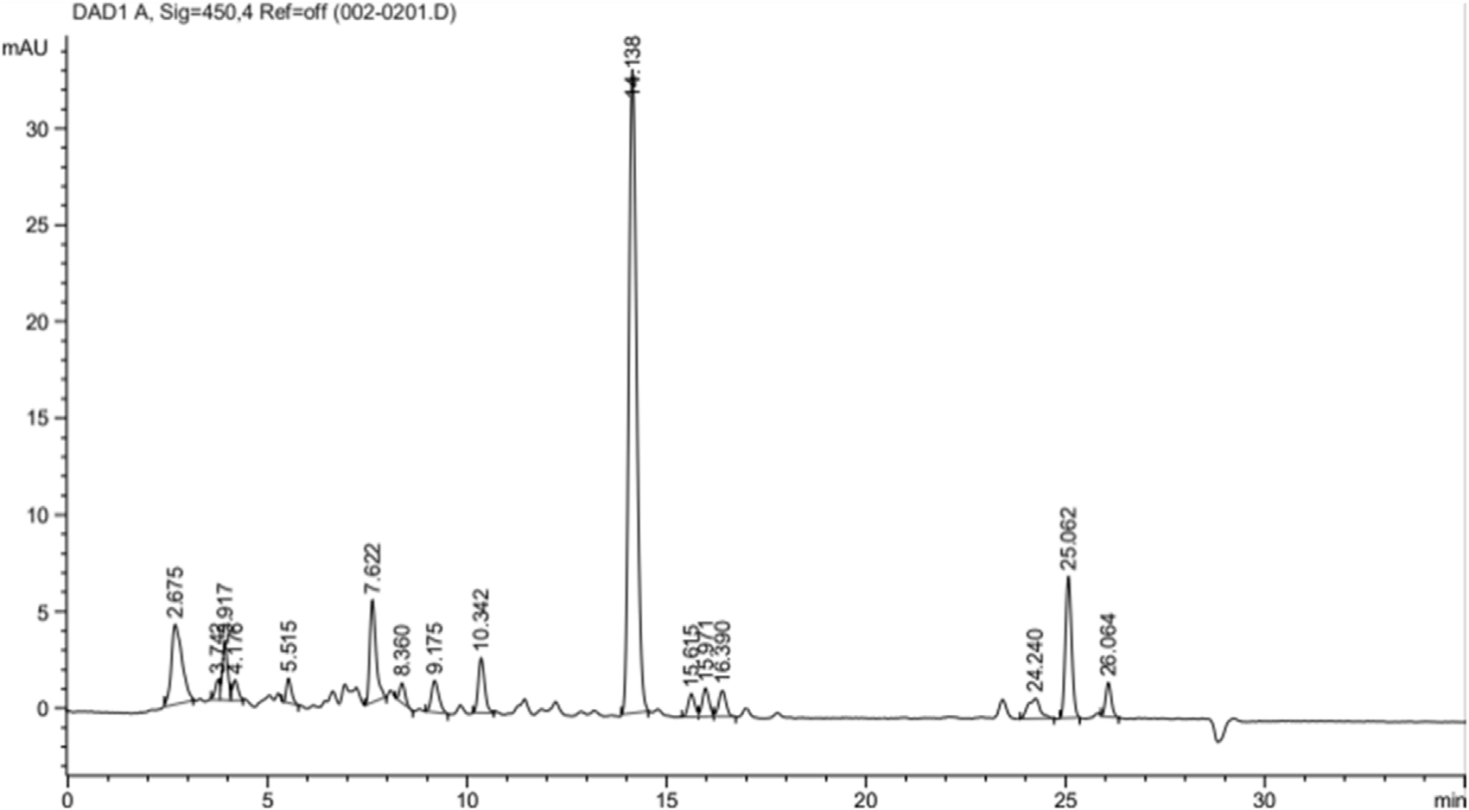
Comprehensive peak characterization trace showing lutein peak from HPLC analyses.

Figure 1 shows the chromatograms of lutein quantification from 1 g of PSPL extract and the peak maxima of the lutein fractions. A variety of peaks have been recorded for the desired lutein compound, with a total quantified lutein content of approximately 20.25% in 1 g of PSPL extract. The maximal elution was recorded at a peak area % of 51.38% at the 14^th^ minute.

### 2.2 Acute toxicity study

#### 2.2.1 Clinical observations

The oral administration of ethanol-extracted *Ipomoea batatas* leaf extract/PSPL did not induce any form of abnormalities in behavioral or eating patterns in the male Sprague–Dawley rats. Moreover, no abnormalities were observed in the gross morphology of the liver, kidney, pancreas, retina, aorta, and heart in the rats administered the highest supplement dose of 2,000 mg/kg. The size, shape, and color of all harvested organs in the AT 2000 group were similar to those in the AC group. All rats survived until the end of the experiments.

#### 2.2.2 Body weight changes

Table 1 shows the changes in body weight in the AT 2000 and AC groups. The body weight gradually increased from day 0, week 1, and week 2 in both the AC and AT 2000 groups. However, the body weight (*p* > 0.05) of the AT 2000 group did not change significantly compared to that of the AC group on day 0, week 1, and week 2.

**TABLE 1:** Changes in body weight in the acute toxicity study. Values are presented as means ± SEM. AC, acute control group; AT 2000, 2,000 mg/kg of PSPL.

	Body weight	
	AC	AT 2000
Day 0	206.60 ± 8.13	210.20 ± 1.53
Week 1	250.40 ± 4.99	262.80 ± 2.03
Week 2	311.25 ± 9.22	301.80 ± 4.33

#### 2.2.3 Calorie and water intake

Table 2 presents the total calorie and water intake for the PSPL acute toxicity study in male Sprague–Dawley rats. The total calorie and water intake did not differ significantly (*p > 0.05*) between the AT 2000 and AC groups.

**TABLE 2:** Total calorie and water intake in the acute toxicity study. Values are presented as means ± SEM. AC, acute control group; AT 2000, 2,000 mg/kg of PSPL.

	AC	AT 2000
Calorie intake (KJ)	475.83 ± 57.96	475.83 ± 22.19
Total water intake (mL)	197.44 ± 11.09	201.60 ± 8.94

#### 2.2.4 Relative organ weight

Table 3 shows the relative organ weights in the AC and AT 2000 groups. The relative organ weight (g) and standardized organ weight (%) did not differ significantly between the AT 2000 and AC groups (*p* > 0.05).

**TABLE 3:** Relative organ weights of SD male rats in the acute toxicity study. Values are expressed as means ± SEM. RpWAT, retroperitoneal; AC, acute control group; AT 2000, 2,000 mg/kg of PSPL.

Organs		
Relative organ weight (g)	AC	AT 2000
Heart	0.94 ± 0.18	0.74 ± 0.17
Liver	10.05 ± 1.23	13.29 ± 1.79
Lungs	1.73 ± 0.28	1.43 ± 0.17
Kidney	2.12 ± 0.31	2.28 ± 0.18
Retina	0.2833 ± 0.15	0.30 ± 0.67
RpWAT	2.22 ± 0.62	1.66 ± 0.19
Visceral fat	1.59 ± 0.34	1.93 ± 0.37
Gonadal fat	1.91 ± 0.79	1.88 ± 0.46
Pancreas	0.89 ± 0.88	0.97 ± 0.07
Spleen	0.74 ± 0.59	0.79 ± 0.14
Percentage per body weight (%)		
Heart	0.32 ± 0.05	0.26 ± 0.07
Liver	3.40 ± 0.28	4.54 ± 0.77
Lungs	0.60 ± 0.12	0.49 ± 0.07
Kidney	0.74 ± 0.13	0.77 ± 0.08
Retina	0.10 ± 0.00	0.10 ± 0.02
RpWAT	0.76 ± 0.04	0.57 ± 0.09
Visceral fat	0.53 ± 0.09	0.65 ± 0.11
Gonadal fat	0.66 ± 0.06	0.63 ± 0.13
Pancreas	0.30 ± 0.02	0.33 ± 0.01
Spleen	0.25 ± 0.01	0.27 ± 0.05

#### 2.2.5 Hematological analysis

Table 4 presents the results of the hematological analysis for the PSPL acute toxicity study in male SD rats. The oral administration of 2,000 mg/kg of PSPL extract led to a significant increase (*p* < 0.05) in MCV and MCHC in the AT 2000 group compared to the AC group. The other measured blood parameters did not differ significantly between the AT 2000 and AC groups (*p* > 0.05).

**TABLE 4:** Effects of PSPL extract on hematological parameters in the acute toxicity study. Values are expressed as means ± SEM. The letters indicate significant differences (*p* < 0.05). Hb, hemoglobin; RBC, red blood cell; RDW, red cell distribution width; PCV, packed cell volume; MCV, mean corpuscular volume; MCH, mean corpuscular hemoglobin; MCHC, mean corpuscular hemoglobin concentration; WBC, white blood cell; AC, acute control group; AT 2000, 2,000 mg/kg of PSPL.

Parameters	AC	AT 2000
Hb (g/dL)	16.4 ± 0.36	16.59 ± 0.81
RBC (x10^12/L)	9.14 ± 0.33	9.28 ± 0.53
RDW (%)	17.17 ± 0.79	17.13 ± 1.10
PCV (%)	59.67 ± 2.33	57.67 ± 3.18
MCV (fL)	65.00 ± 0.58^a^	62.00 ± 0^b^
MCH (pg)	17.67 ± 0.33	18.00 ± 0
MCHC (g/dL)	27.67 ± 0.33^a^	29.33 ± 0.33^b^
WBC (x10^9/L)	13.67 ± 1.03	16.70 ± 1.76
Neutrophils (x10^9/L)	2.07 ± 0.37	2.27 ± 0.41
Lymphocytes (x10^9/L)	9.97 ± 0.73	13.10 ± 1.69
Monocytes (x10^9/L)	1.40 ± 0.12	1.03 ± 0.37
Eosinophils (x10^9/L)	0.19 ± 0.04	0.19 ± 0.18
Basophils (x10^9/L)	0.07 ± 0.03	0.10 ± 0.00
Platelet (x10^9/L)	1,235.00 ± 124.82	1,390.67 ± 183.97

#### 2.2.6 Blood biochemistry analysis

Table 5 presents the results of the blood biochemistry analysis in the PSPL acute toxicity study in male Sprague–Dawley rats. The data showed no significant difference (*p* > 0.05) in the renal and liver profiles between the AT 2000 and AC groups.

**TABLE 5:** Effect of PSPL extracts on blood biochemistry parameters in the SD male acute toxicity study. Values are expressed as means ± SEM. ALP, alkaline phosphatase; AST, aspartate transaminase; ALT, alanine transaminase; GGT, gamma-glutamyl transferase; AC, acute control group; AT 2000, 2,000 mg/kg of PSPL.

Parameters	AC	AT 2000
Renal function test		
Sodium (mmol/L)	145.00 ± 1.00	145.33 ± 0.88
Potassium (mmol/L)	10 ± 0	9.5 ± 0.5
Chloride (mmol/L)	97.00 ± 1.53	100.67 ± 1.45
Urea (mmol/L)	8.30 ± 0.21	7.60 ± 0.58
Creatinine (mmol/L)	51.00 ± 4.51	44.33 ± 4.41
Liver Function Test		
Total protein (g/L)	69.33 ± 0.67	69.33 ± 2.96
Albumin (g/L)	40.33 ± 1.20	41.00 ± 0.58
Globulin (g/L)	29.00 ± 0.58	28.33 ± 2.40
Albumin–globulin ratio	1.39 ± 0.07	1.46 ± 0.01
ALP (U/L)	371.00 ± 16.01	290.00 ± 52.27
AST (U/L)	136.00 ± 7.94	144.67 ± 29.49
ALT (U/L)	86.00 ± 8.89	92.00 ± 25.03
GGT (U/L)	1.00 ± 0.00	1.00 ± 0.00
Total bilirubin (µmol/L)	1.00 ± 0.00	1.00 ± 0.00

#### 2.2.7 Histopathological analysis

**FIGURE 2:**
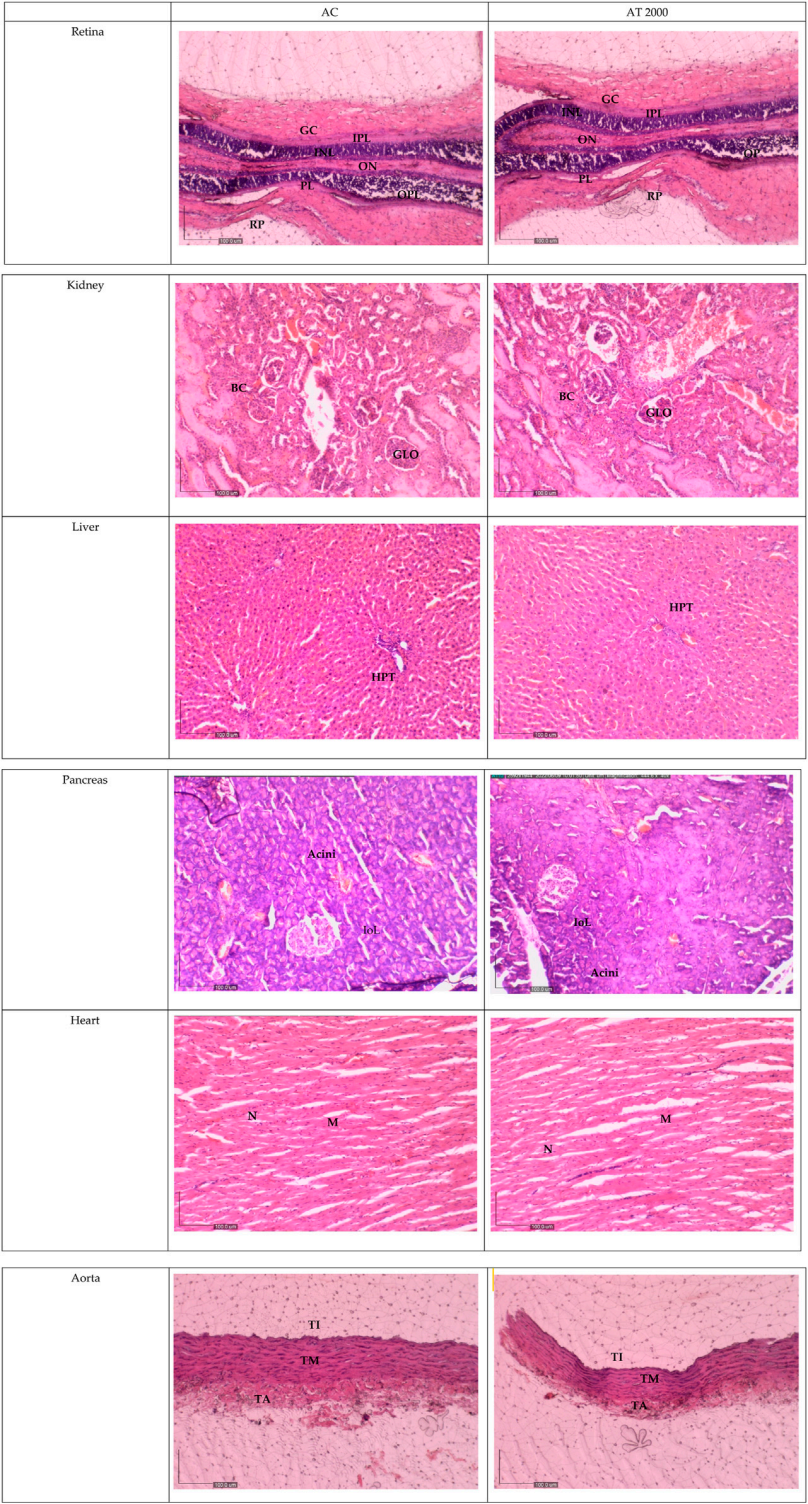
Histology section of the AT 2000 and AC groups. No abnormalities in the histological sections of the retina, kidney, liver, pancreas, heart, and aorta of the AT 2000-treated group were observed compared to the AC group. GCL, ganglion cell layer; IPL, inner plexiform layer; INL, inner nuclear layer; OPL, outer plexiform layer; ONL, outer nuclear layer; PL, photoreceptor layer; RPE, retinal pigmented epithelium monolayer; BC, Bowman’s capsule; GLO, glomerulus; iOL, islet of Langerhans; HPL, hepatic portal triad; TI, tunica intima; TM, tunica media; TA, tunica adventitia; N, nucleus; M, myocardium; AC, acute control group; AT 2000, 2,000 mg/kg of PSPL.

Figure 2 shows the histological sections of the retina, kidney, pancreas, liver, heart, and aorta. No pathological abnormalities were observed, and the AT 2000 group showed similar morphological and histological features compared to those in the AC group. The retina showed normal features of the ganglion cell layer (GCL), inner plexiform layer (IPL), inner nuclear layer (INL), outer plexiform layer (OPL), outer nuclear layer (ONL), photoreceptor layer (PL), and retinal pigmented epithelium monolayer (RPE). The kidney histology showed the presence of Bowman’s capsule (BC) and glomerulus (GLO). The normal histology features of the islet of Langerhans (ioL) and Acini were observed in the pancreas, and normal strands of the hepatic portal triad (HPL) were visible in liver histology. The histological sections showed the presence of the aorta tunica intima (TI), tunica media (TM), and tunica adventitia (TA). The heart structure shows the nucleus (N) and myocardium (M).

### 2.3 Subacute toxicity study (28-day repeated dose study)

#### 2.3.1 Clinical observation

The oral administration of PSPL extract at various concentrations (ST 50, ST 250, ST 500, and ST 1000) for subacute toxicity study for 28 days and SST 1000 for 28 days of treatment followed by an additional 14-day observation without any treatment did not induce any abnormalities in behavior patterns or physical condition. All rats showed normal responses to touch and in salivation, hair coat, eye color, sleeping pattern, and grip strength. No locomotor dysfunction, convulsions, or tremors were recorded in the rats. All rats survived until the end of the experiment.

#### 2.3.2 Body weight changes

Table 6 shows the changes in body weight in the subacute toxicity groups recorded for 28 days, and the satellite group recorded for 42 consecutive days. The body weights did not differ significantly between the ST 50, ST 250, ST 500, ST 1000, and SC groups at day 0 and weeks 1, 2, 3, and 4 (*p* > 0.05). The satellite group analysis showed no significant differences in body weight between the SST and SSC groups on day 0 and weeks 1, 2, 3, 4, 5, and 6 (*p* > 0.05).

**TABLE 6:** Weekly body weight changes in the subacute toxicity study. Values are expressed as means ± SEM.

	Groups for the subacute toxicity study^a^					Satellite group for the subacute toxicity study^b^	
Body weight (g)	SC	ST 50	ST 250	ST 500	ST 1000	SSC	SST 1000
Day 0	242.17 ± 15.10	246.67 ± 5.30	242.17 ± 16.98	236.5 ± 12.99	243.67 ± 14.70	239.33 ± 10.68	234.17 ± 11.26
Week 1	285.00 ± 22.11	291.00 ± 11.35	284.33 ± 20.54	282.50 ± 10.29	271.67 ± 13.73	270.33 ± 14.67	272.00 ± 12.81
Week 2	324.00 ± 21.29	319.33 ± 15.66	313.83 ± 17.87	305.00 ± 15.33	302.83 ± 11.82	270.67 ± 16.70	306.67 ± 14.96
Week 3	348.50 ± 25.67	344.33 ± 19.45	333.67 ± 17.43	327.67 ± 17.21	322.83 ± 11.56	302.00 ± 21.71	329.50 ± 16.52
Week 4	365.67 ± 26.12	357.17 ± 20.31	349.33 ± 14.88	344.17 ± 16.54	335.17 ± 12.26	323.00 ± 22.39	344.00 ± 18.47
Week 5						341.67 ± 26.27	361.33 ± 20.00
Week 6						362.83 ± 24.04	386.50 ± 21.44

^a^ Groups for the subacute toxicity study.

^b^ Satellite groups for the subacute toxicity study.

SC, subacute control; ST 50, 50 mg/kg of PSPL; ST 250, 250 mg/kg of PSPL; ST 500, 500 mg/kg of PSPL; ST 1000, 1,000 mg/kg of PSPL, SSC, subacute satellite control group; SST, subacute satellite group.

#### 2.3.3 Total calorie and water intake

Table 7 presents the weekly calorie and 24-h water intakes for the subacute toxicity and satellite groups. The calorie and water intake did not differ significantly between the ST 50, ST 250, ST 500, ST 1000, and SC groups (*p* > 0.05). The satellite group analysis showed no significant differences in calorie and water intake between the SST 1000 and SSC groups (*p* > 0.05).

**TABLE 7:** Calorie and water intake in the subacute toxicity study. Values are presented as means ± SEM.

	Groups for the subacute toxicity study^a^					Satellite group for the subacute toxicity study^b^	
	SC	ST 50	ST 250	ST 500	ST 1000	SSC	SST 1000
Calorie intake (KJ)	521.56 ± 10.52	507.27 ± 17.74	503.7 ± 10.10	489.41 ± 42.97	478.69 ± 5.83	221.67 ± 9.53	291.67 ± 38.10
Total water intake (mL)	365.67 ± 18.47	357.17 ± 14.36	349.33 ± 10.52	344.17 ± 11.70	335.17 ± 8.67	221.67 ± 9.53	291.67 ± 38.1

^a^ Groups for the subacute toxicity study.

^b^ Satellite groups for the subacute toxicity study.

SC, subacute control; ST 50, 50 mg/kg of PSPL; ST 250, 250 mg/kg of PSPL; ST 500, 500 mg/kg of PSPL; ST 1000, 1,000 mg/kg of PSPL, SSC, subacute satellite control group; SST, subacute satellite group.

#### 2.3.4 Relative organ weights

Table 8 shows the relative organ weights in the subacute toxicity and satellite groups. The relative and standardized organ weights (%) did not differ significantly between the ST 50, ST 250, ST 500, ST 1000, and SC groups (*p* > 0.05). The satellite group analysis showed no significant difference in relative and standardized organ weights (%) in the SST 1000 group compared to the SSC group (*p* > 0.05).

**TABLE 8:** Relative organ weights of rats treated with PSPL extract in the subacute toxicity study. Values are expressed as means ± SEM.

Organs	Groups for the subacute toxicity study^a^						Satellite group for the subacute toxicity study^b^	
	SC	ST 50	ST 250	ST 500		ST 1000	SSC	SST 1000
Relative organ weight (g)								
Heart	1.26 ± 0.07	1.17 ± 0.11	1.14 ± 0.03	1.31 ± 0.05		1.22 ± 0.06	1.02 ± 0.07	1.17 ± 0.04
Liver	11.60 ± 0.67	11.95 ± 0.53	12.41 ± 0.63	11.97 ± 0.90		10.51 ± 0.80	11.73 ± 0.85	11.37 ± 0.62
Lungs	2.21 ± 0.24	1.98 ± 0.12	1.92 ± 0.10	2.15 ± 0.26		2.15 ± 0.14	1.99 ± 0.12	2.09 ± 0.14
Kidney	2.58 ± 0.06	2.52 ± 0.12	2.56 ± 0.12	2.48 ± 0.17		2.55 ± 0.09	2.43 ± 0.16	2.54 ± 0.10
Retina	0.40 ± 1.74	0.35 ± 0.04	0.31 ± 0.03	0.29 ± 0.04		0.34 ± 0.03	0.53 ± 0.20	0.37 ± 0.05
RpWAT	5.45 ± 0.52	3.61 ± 0.82	3.48 ± 0.41	3.57 ± 0.59		3.46 ± 0.69	3.12 ± 0.49	3.65 ± 0.52
Visceral fat	2.33 ± 0.15	1.26 ± 0.37	1.86 ± 0.20	1.70 ± 0.37		1.79 ± 0.36	1.55 ± 0.20	1.41 ± 0.19
Gonadal fat	3.70 ± 0.57	3.72 ± 0.67	2.95 ± 0.61	3.46 ± 0.51		3.43 ± 0.20	2.90 ± 0.31	4.00 ± 0.42
Pancreas	1.10 ± 0.12	1.22 ± 0.09	0.99 ± 0.08	0.98 ± 0.09		1.15 ± 0.09	1.27 ± 0.41	0.91 ± 0.08
Spleen	0.81 ± 0.05	0.75 ± 0.10	0.65 ± 0.03	0.67 ± 0.06		0.75 ± 0.05	0.69 ± 0.07	0.66 ± 0.04
Percentage per body weight (%)								
Heart	0.35 ± 0.01	0.33 ± 0.03	0.33 ± 0.01	0.39 ± 0.02		0.37 ± 0.02	0.31 ± 0.02	0.32 ± 0.01
Liver	3.26 ± 0.17	3.40 ± 0.09	3.61 ± 0.22	3.49 ± 0.17		3.15 ± 0.15	3.23 ± 0.11	3.10 ± 0.17
Lungs	0.62 ± 0.07	0.57 ± 0.04	0.56 ± 0.02	0.63 ± 0.07		0.65 ± 0.05	0.57 ± 0.05	0.57 ± 0.03
Kidney	0.73 ± 0.04	0.72 ± 0.02	0.74 ± 0.03	0.73 ± 0.04		0.77 ± 0.02	0.68 ± 0.02	0.69 ± 0.02
Retina	0.11 ± 0.01	0.10 ± 0.01	0.09 ± 0.01	0.09 ± 0.01		0.10 ± 0.01	0.09 ± 0.01	0.10 ± 0.01
RpWAT	1.57 ± 0.19	1.00 ± 0.22	1.01 ± 0.11	1.03 ± 0.14		1.04 ± 0.21	0.93 ± 0.20	0.98 ± 0.13
Visceral fat	0.66 ± 0.06	0.36 ± 0.10	0.54 ± 0.05	0.49 ± 0.10		0.53 ± 0.10	0.43 ± 0.07	0.39 ± 0.06
Gonadal fat	1.07 ± 0.17	1.04 ± 0.16	0.88 ± 0.1	1.00 ± 0.12		1.04 ± 0.06	0.81 ± 0.09	1.08 ± 0.10
Pancreas	1.07 ± 0.17	1.04 ± 0.16	0.88 ± 0.18	1.00 ± 0.12		1.04 ± 0.06	0.26 ± 0.03	0.25 ± 0.03
Spleen	0.23 ± 0.01	0.21 ± 0.03	0.19 ± 0.01	0.20 ± 0.02		0.23 ± 0.01	0.18 ± 0.00	0.18 ± 0.01

^a^ Groups for the subacute toxicity study.

^b^ Satellite groups for the subacute toxicity study.

RpWAT, retroperitoneal white adipose tissue; SC, subacute control; ST 50, 50 mg/kg of PSPL; ST 250, 250 mg/kg of PSPL; ST 500, 500 mg/kg of PSPL; ST 1000, 1,000 mg/kg of PSPL, SSC, subacute satellite control group; SST, subacute satellite group.

#### 2.3.5 Hematological analysis

Table 9 presents the hematological parameters in the subacute toxicity and satellite groups. The hematological parameters did not differ significantly between the ST 50, ST 250, ST 500, ST 1000, and SC groups (*p* > 0.05). The satellite group analysis showed no significant differences in hematological parameters between the SST 1000 and SSC groups (*p* > 0.05).

**TABLE 9:** Hematological effects of different treatment doses of PSPL extract on male Sprague–Dawley rats in the subacute toxicity study. Values are expressed as means ± SEM.

	Groups for the subacute toxicity study^9^					Satellite group for the subacute toxicity study^b^	
	SC	ST 50	ST 250	ST 500	ST 1000	SSC	SST 1000
Hb (g/dL)	15.50 ± 0.55	15.75 ± 0.36	16.48 ± 0.42	15.60 ± 0.35	15.33 ± 0.29	16.27 ± 0.49	17.08 ± 0.61
RBC (x10^12/L)	8.91 ± 0.33	9.06 ± 0.22	9.54 ± 0.30	8.89 ± 0.13	8.97 ± 0.18	9.59 ± 028	9.92 ± 0.35
RDW (%)	17.45 ± 0.53	17.57 ± 0.37	17.43 ± 0.85	17.12 ± 0.33	17.43 ± 0.21	18.02 ± 0.35	18.68 ± 0.47
PCV (%)	55.67 ± 1.96	56.00 ± 1.48	58.33 ± 1.54	54.50 ± 1.50	52.50 ± 1.02	58.00 ± 1.65	58.50 ± 1.95
MCV (fL)	62.33 ± 0.84	61.83 ± 1.01	61.50 ± 0.10	61.17 ± 1.25	58.50 ± 0.72	60.50 ± 0.62	58.83 ± 0.54
MCH (pg)	17.67 ± 0.21	17.33 ± 0.21	17.50 ± 0.22	17.50 ± 0.22	17.17 ± 0.17	17.00 ± 0.26	17.17 ± 0.31
MCHC (g/dL)	28.00 ± 0.26	28.33 ± 0.32	28.17 ± 0.17	28.50 ± 0.29	29.00 ± 0.00	28.33 ± 0.33	29.33 ± 0.33
WBC (x10^9/L)	19.62 ± 3.16	19.30 ± 2.24	17.03 ± 2.10	20.57 ± 1.31	19.12 ± 0.89	19.78 ± 1.43	20.62 ± 1.74
Neutrophils (x10^9/L)	3.93 ± 1.06	3.65 ± 0.24	3.55 ± 0.46	4.87 ± 0.39	3.38 ± 0.65	3.58 ± 0.36	2.67 ± 0.27
Lymphocytes (x10^9/L)	13.52 ± 1.85	14.65 ± 1.97	12.28 ± 1.77	34.22 ± 19.59	14.83 ± 0.94	14.70 ± 1.11	16.73 ± 1.56
Monocytes (x10^9/L)	1.88 ± 1.04	0.72 ± 0.15	0.95 ± 0.35	0.97 ± 0.13	0.62 ± 0.08	1.15 ± 0.44	0.93 ± 0.24
Eosinophils (x10^9/L)	0.17 ± 0.04	0.19 ± 0.02	0.15 ± 0.03	0.20 ± 0.03	0.23 ± 0.02	0.23 ± 0.04	0.18 ± 0.04
Basophils (x10^9/L)	0.10 ± 0.03	0.07 ± 0.02	0.08 ± 0.02	0.10 ± 0.00	0.10 ± 0.00	0.10 ± 0.00	0.10 ± 0.00
Platelet (x10^9/L)	1,053.50 ± 45.11	1,231.67 ± 73.97	1,205.50 ± 45.70	1,247.00 ± 96.80	1,158.50 ± 64.27	1,215.67 ± 98.12	1,265.83 ± 80.51

^a^ Group for the subacute toxicity study.

^b^ Satellite groups for the subacute toxicity study.

Hb, hemoglobin; RBC, red blood cell; RDW, red cell distribution width; PCV, packed cell volume; MCV, mean corpuscular volume; MCH, mean corpuscular hemoglobin; MCHC, mean corpuscular hemoglobin concentration; WBC, white blood cell; SC, subacute control; ST 50, 50 mg/kg of PSPL; ST 250, 250 mg/kg of PSPL; ST 500, 500 mg/kg of PSPL; ST 1,000, 1,000 mg/kg of PSPL, SSC, subacute satellite control group; SST, subacute satellite group.

#### 2.3.6 Blood biochemistry analysis

Table 10 shows the blood biochemistry data for the subacute toxicity and satellite groups. The blood biochemical parameters did not differ significantly between the ST 50, ST 250, ST 500, ST 1000, and SC groups (*p* > 0.05). The satellite group analysis showed no significant differences in blood biochemical parameters between the SST 1000 and SSC groups (*p* > 0.05).

**TABLE 10:** Effects of PSPL extract on biochemistry analysis in the subacute toxicity studies. Values are expressed as means ± SEM.

	Groups for the subacute toxicity study^a^					Satellite group for subacute toxicity study^b^	
	SC	ST 50	ST 250	ST 500	ST 1000	SSC	SST 1000
Renal function test							
Sodium (mmol/L)	144.50 ± 0.56	144.50 ± 1.95	146.00 ± 1.03	146.00 ± 0.82	143.00 ± 0.49	144.80 ± 0.20	145.17 ± 0.79
Potassium (mmol/L)	8.43 ± 1.74	10.48 ± 0.29	9.65 ± 0.79	10.10 ± 0.49	10.64 ± 0.36	11.00 ± 0.00	10.78 ± 0.22
Chloride (mmol/L)	98.50 ± 0.56	100.00 ± 0.82	97.25 ± 0.48	99.00 ± 0.77	98.40 ± 0.93	97.00 ± 0.71	100.67 ± 1.43
Urea (mmol/L)	7.18 ± 0.33	7.70 ± 0.60	8.70 ± 0.89	8.55 ± 0.52	6.88 ± 0.24	8.60 ± 0.15	7.73 ± 0.61
Creatinine (mmol/L)	57.50 ± 3.43	55.33 ± 2.87	60.25 ± 1.60	53.33 ± 1.80	52.40 ± 2.58	54.60 ± 2.25	51.33 ± 4.62
Liver function test							
Total protein (g/L)	70.00 ± 0.82	71.17 ± 0.98	76.25 ± 0.48	70.17 ± 1.17	69.40 ± 0.93	74.00 ± 0.55	75.33 ± 1.28
Albumin (g/L)	38.83 ± 0.87	40.00 ± 0.68	42.50 ± 0.50	39.83 ± 1.08	36.40 ± 0.40	40.60 ± 0.51	41.83 ± 0.48
Globulin (g/L)	31.17 ± 1.10	31.17 ± 1.01	33.75 ± 0.25	30.33 ± 0.88	33.00 ± 1.00	33.40 ± 0.51	33.50 ± 0.96
Albumin–globulin ratio	1.26 ± 0.06	1.29 ± 0.05	1.26 ± 0.02	1.32 ± 0.06	1.09 ± 0.04	1.22 ± 0.03	1.25 ± 0.03
ALP (U/L)	225.00 ± 17.25	211.17 ± 13.96	254.67 ± 18.08	212.67 ± 14.99	214.60 ± 27.78	254.20 ± 32.93	186.83 ± 18.37
AST (U/L)	118.83 ± 14.43	149.33 ± 17.80	115.75 ± 14.52	126.00 ± 6.79	128.60 ± 14.02	156.00 ± 11.97	115.83 ± 12.78
ALT (U/L)	71.50 ± 9.83	77.17 ± 6.46	86.25 ± 11.60	86.17 ± 8.11	86.80 ± 10.17	108.40 ± 11.63	77.33 ± 9.02
GGT (U/L)	0.67 ± 0.21	1.17 ± 0.31	1.25 ± 0.25	1.00 ± 0.00	1.20 ± 0.20	1.00 ± 0.00	1.00 ± 0.00
Total bilirubin (µmol/L)	1.00 ± 0.00	1.00 ± 0.00	1.00 ± 0.00	1.00 ± 0.00	1.00 ± 0.00	1.00 ± 0.00	1.00 ± 0.00

^a^ Groups for the subacute toxicity study.

^b^ Satellite groups for the subacute toxicity study.

ALP, alkaline phosphatase; AST, aspartate transaminase; ALT, alanine transaminase; GGT, gamma-glutamyl transferase; SC, subacute control; ST 50, 50 mg/kg of PSPL; ST 250, 250 mg/kg of PSPL; ST 500, 500 mg/kg of PSPL; ST 1000, 1,000 mg/kg of PSPL, SSC, subacute satellite control group; SST, subacute satellite group.

#### 2.3.7 Histopathological analysis

**FIGURE 3:**
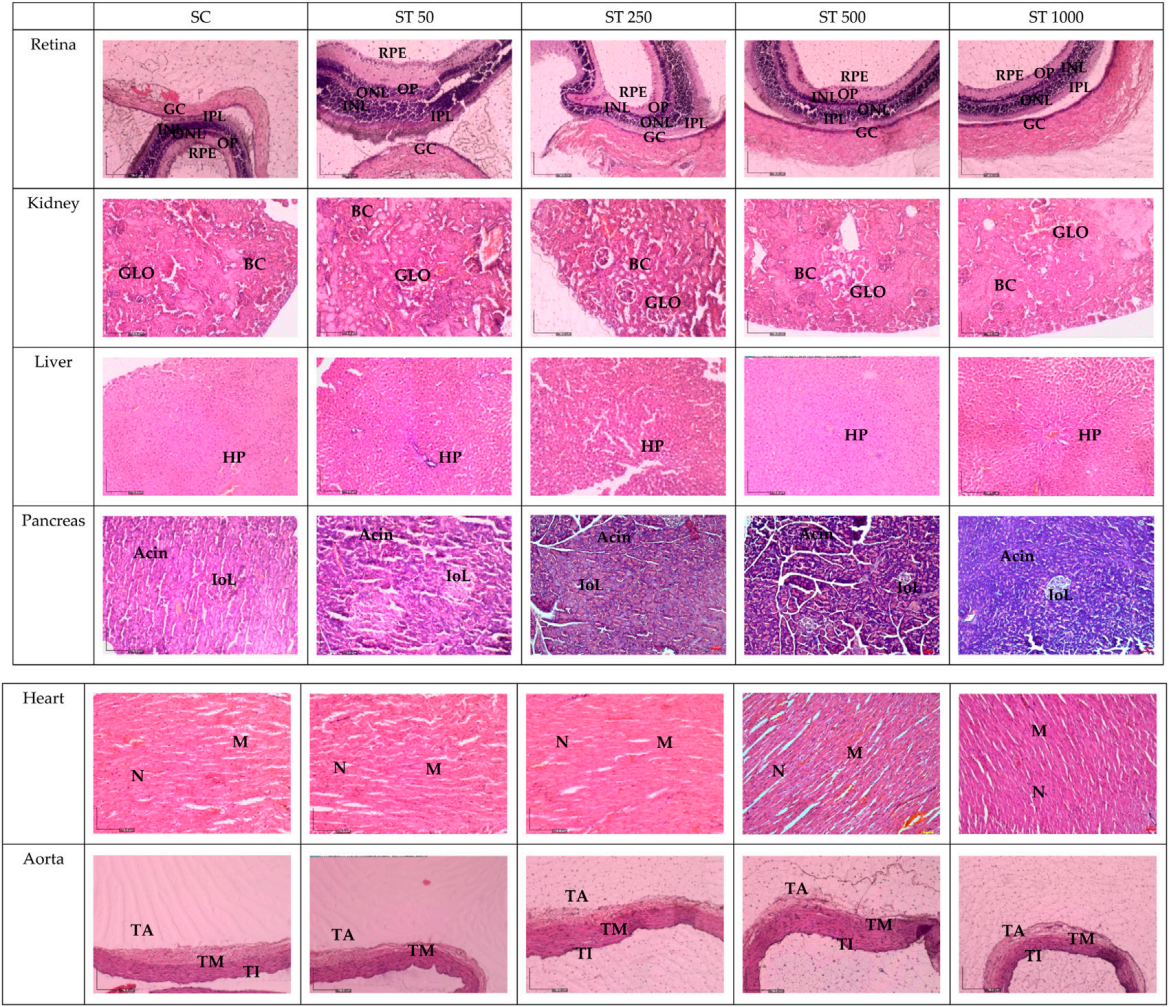
Histology sections in the subacute studies. No abnormalities in the histological sections of the retina, kidney, liver, pancreas, heart, and aorta of the ST 50, ST 250, ST 500, and ST 1000-treated groups were observed compared to the SC group. GCL, ganglion cell layer; IPL, inner plexiform layer; INL, inner nuclear layer; OPL, outer plexiform layer; ONL, outer nuclear layer; PL, photoreceptor layer; RPE, retinal pigmented epithelium monolayer; BC, Bowman’s capsule; GLO, glomerulus; iOL, islet of Langerhans; HPL, hepatic portal triad; TI, tunica intima; TM, tunica media; TA, tunica adventitia; N, nucleus; M, myocardium; SC, subacute control; ST 50, 50 mg/kg of PSPL; ST 250, 250 mg/kg of PSPL; ST 500, 500 mg/kg of PSPL; ST 1000, 1,000 mg/kg of PSPL.

**FIGURE 4:**
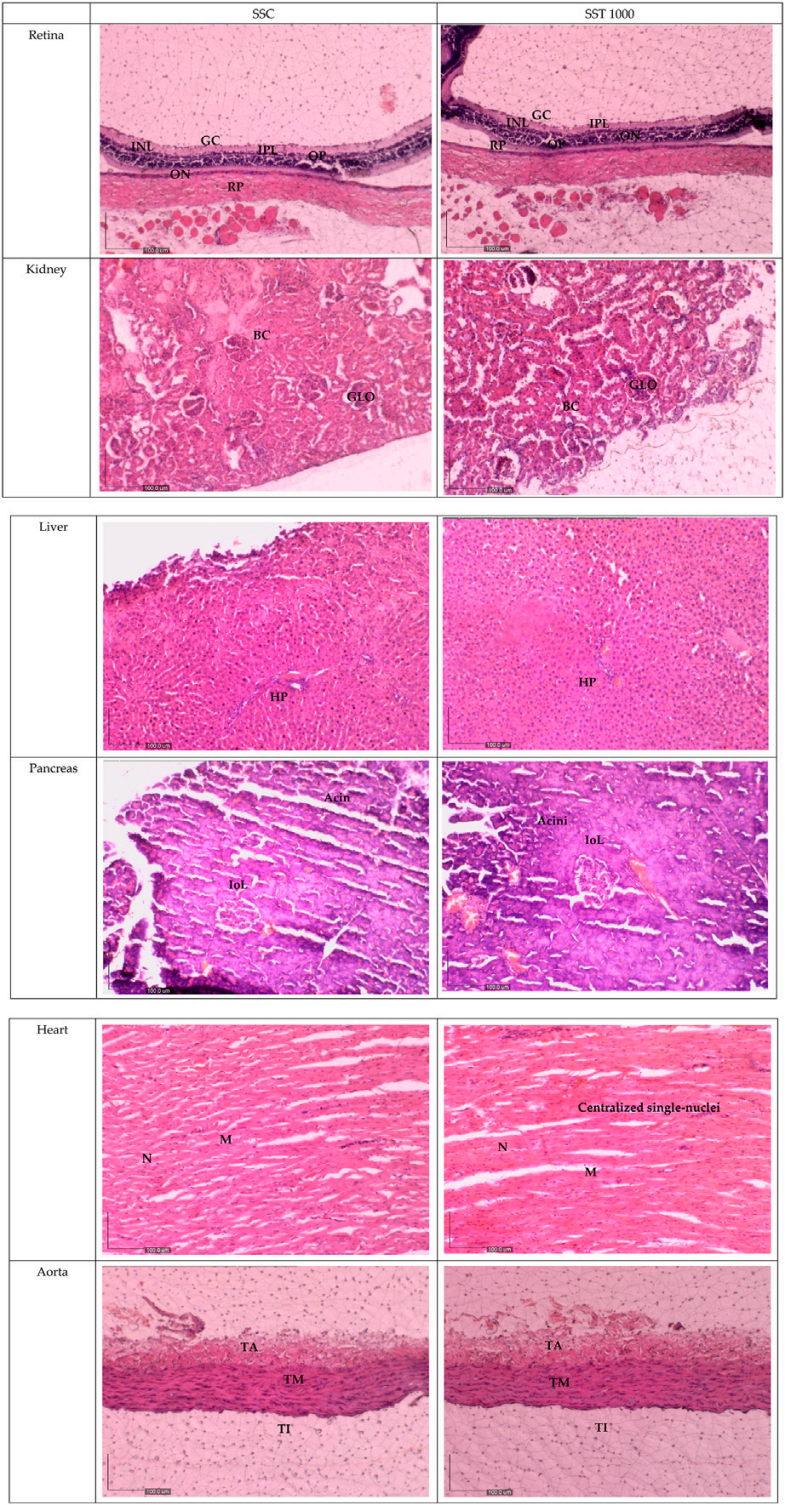
Histology sections for the satellite groups in the subacute studies. No abnormalities in the histological sections of the retina, kidney, liver, pancreas, heart, and aorta of the SST 1000-treated group were observed compared to the SSC group. GCL, ganglion cell layer; IPL, inner plexiform layer; INL, inner nuclear layer; OPL, outer plexiform layer; ONL, outer nuclear layer; PL, photoreceptor layer; RPE, retinal pigmented epithelium monolayer; BC, Bowman’s capsule; GLO, glomerulus; iOL, islet of Langerhans; HPL, hepatic portal triad; TI, tunica intima; TM, tunica media; TA, tunica adventitia; N, nucleus; M, myocardium; SSC, subacute satellite control group; SST, subacute satellite group.

Figure 3 shows histological sections of the retina, kidney, pancreas, liver, heart, and aorta. No pathological abnormalities were observed in the ST 50, ST 250, ST 500, and ST 1000 groups, which showed similar morphological and histological features as the SC group. Similarly, the satellite group showed no histological abnormalities in the SST 1000 and SST 1000 groups, with similar features as the SSC group (Figure 4). Histological analysis of the retina showed normal features of the ganglion cell layer (GCL), inner plexiform layer (IPL), inner nuclear layer (INL), outer plexiform layer (OPL), outer nuclear layer (ONL), and retinal pigmented epithelium monolayer (RPE). The kidney histology showed the presence of Bowman’s capsule (BC) and glomerulus (GLO). Normal histological features of the islet of Langerhans (ioL) and Acini were seen in the pancreas, while normal strands of the hepatic portal triad (HPL) were visible in liver histology. The histological sections showed the aorta tunica intima (TI), tunica media (TM), and tunica adventitia (TA). The heart structure showed the nucleus (N) and myocardium (M).

## 3 Discussion

PSPL is traditionally known for its beneficial therapeutic effects (Akhtar et al., 2018). However, it remains underutilized due to safety concerns. The results of the present study showed that the PSPL extract contained the highest % of lutein compound, approximately 20.25%, compared to other leafy vegetables (Sahli et al., 2015). The study is divided into two phases. The first phase focused on the phytochemical analysis of lutein percentage, while the second phase focused on the toxicological evaluation of PSPL extract in male Sprague–Dawley rats. The safety analysis of PSPL extracts in SD male rats in the first phase was further categorized into acute or subacute studies. In the acute study, a single maximum dose of 2,000 mg/kg per body weight did not induce any side effects or behavioral or sleep abnormalities. All rats survived until necropsy. Similar findings were observed in the subacute study, including satellite groups comprising four different doses (ST 50, ST 250, ST 500, and ST 1000) of PSPL. Neither the acute nor the subacute studies revealed any drastic or significant changes in body weight, organ weight, calorie intake, or water consumption from the beginning to the end of the assessment period.

Due to their roles in metabolism and excretion, the liver and kidneys are the most important organs to rule out toxicity (Peter et al., 2018). Gross examination and histopathological changes indicate toxicity (Zakaria et al., 2011). However, the similar histological features of the kidney and liver between the AT 2000 and AC groups; between the ST 50, ST 250, ST 500, and ST 1000 groups and the SC group; and between the SST 1000 and SSC groups further proves the safety of up to 2,000 mg/kg of PSPL extract. The gross examinations of the liver and kidney revealed no changes in the treated groups compared to the control group. In addition, H&E staining of the retina, pancreas, heart, and aorta to assess any abnormalities showed that the treated group was indistinguishable from the control groups in both acute and subacute studies. Moreover, the liver and kidney profile assessments of the blood biochemistry appeared within normal ranges for all assessed parameters, specifically ALP, AST, and ALP, which are also biomarkers of liver toxicity (Meunier and Larrey, 2019), thus further proving that the PSPL extract was safe for consumption up to a dose of 2,000 mg/kg.

Changes in hematological parameters are an indicator of the emergence of toxicity (Kamisan et al., 2012). However, the administration of 2,000 mg/kg of PSPL extract caused a significant increase in MCV and MCHC compared to AC. Increased MCV suggests macrocytic effects, while increased MCHC is associated with hyperchromic effects. Such conditions are usually associated with increased RBC rigidity (Tempelhoff et al., 2016). However, given the normal levels of other blood parameters (RBC, WBC, HB, platelet, RDW, and MCH), the possibilities of macrocytic and hyperchromic conditions were ruled out. The slight increase in MCV and MCHC in the AT 2000 group could be due to incidental instead of treatment-related effects. Moreover, MCV and MCHC usually increase with age (Tempelhoff et al., 2016). Thus, slight increases in MCH and MCHC are not concerning. Hence, the findings regarding the changes in body weight, organ weight, calorie intake, water consumption, blood biochemistry assessment, hematological analysis, and histopathological assessments in the acute and subacute studies demonstrated that the administration of up to 2,000 mg/kg/day of PSPL extract is a tolerable dosage.

## 4 Conclusion

In the present study, the PSPL extract showed the highest concentration of lutein of approximately 20.25%, which is the highest among natural roots. The therapeutic effects of the extract in biomedical applications were further analyzed. No signs of toxicity in the blood components or serum biochemical measures were observed in either the acute and subacute studies. The toxicological evaluation showed no abnormalities in the tissue histology nor gross morphology of the retina, kidney, liver, pancreas, aorta, and heart, indicating that the administration of up to 2,000 mg/kg/day of PSPL was well-tolerated. However, further assessments of the genotoxicity, compound toxicity, and subchronic and chronic toxicity are needed before proceeding with clinical trials, and it is highly recommended to test the compound in various age groups before PSPL use in clinical prescriptions.

## 5 Materials and methods

### 5.1 Plant species collection and confirmation

Fresh PSPL was obtained from a commercial sweet potato farm located at Sungai Pelek, Sepang, Selangor, Malaysia. The leaves were cleaned and sent to Herbarium Biodiversity Unit, Universiti Putra Malaysia for confirmation (voucher code: MFI 0188/20).

### 5.2 Ethanol extraction of *Ipomoea batatas* leaves

The PSPL leaves were cleaned with running tap water to remove any foreign material. About 20 g of PSPL leaves was soaked in 200 mL of 80% ethanol in a conical flask. The mixture was then placed in an orbital incubator shaker for 24 h at 150 rpm at room temperature. The supernatant was then collected and filtered using Whatman N°1 paper. The process was repeated three times to obtain the maximum yield. The filtrates from each extraction were then combined and evaporated using a rotary evaporator at 48°C. The obtained crude extract was then mixed with maltodextrin in a 1:1 ratio and oven-dried overnight (Fu et al., 2016). The final extracted powder was then weighed and stored at −20°C until further use.

### 5.3 HPLC quantification of the active compounds

High-performance liquid chromatography (HPLC) estimation of the lutein compound was performed in an Agilent 1200 Infinity instrument (Agilent Technology, United States) equipped with a DAD detector, an autosampler, a column heater, and a Welchrom^®^ C18 (4.67 mm ID, 250 mm, 5 μm particle size) column. The mobile phase consisted of two solvents: (A) 0.140 g of anhydrous potassium dihydrogen orthophosphate (KH2PO) dissolved in 900 mL HPLC-grade water with the addition of 0.5 mL orthophosphoric acid, made up to 1,000 mL with water and filtered through a 0.45-μ membrane filter, and degassed in a sonicator for 3 min and (B) acetonitrile (HPLC-grade). With a flow rate of 1.5 mL/min, gradient elution was performed using the two solvents at a detection wavelength of 348 nm. A 20 μL sample volume was injected into the system by the autosampler. About 10.97 mg of the isolated esculetin was dissolved in 10 mL of ethanol, while 6.26 mg PSPL extract was dissolved in 10 mL of ethanol. HPLC was then performed for both the standard and sample (Makwana and Pandya, 2020).

### 5.4 Experimental animals for the toxicity studies

All animal-related protocols for the toxicity studies were performed according to the guideline of the Institutional Animal Care and Use Committee (IACUC) of UPM (ethical code UPM/IACUC/AUP-R070/2020). A total of 54 adult male Sprague–Dawley rats weighing 150–200 g were used. All rats were subjected to acclimatization for the first week at a controlled temperature room (23°C–25°C) and humidity (55%–60%) and with a 12/12 h light/dark cycle. All rats were fed standard rat pellets *ad libitum*. Changes in body weight and 24-h water intake were measured weekly (Bahari et al., 2020).

### 5.5 Acute toxicity study

An acute toxicity study was carried out according to the protocol described in OECD 423. For acute toxicity assessment, the rats were divided into two groups (n = 3). The acute control group (AC) was administered normal saline through bottle feeding, while the treatment group was administered 2,000 mg/kg of PSPL (AT 2000) extract orally for 2 weeks. All rats were under constant observation for the entire treatment duration. The rats were observed for any abnormalities until euthanasia. All rats were euthanized with carbon dioxide (CO_2_) overdose, and their blood was collected in an EDTA tube for biochemical analysis. Organs such as the liver, kidney, pancreas, retina, aorta, and heart were harvested, weighed, and kept in 10% neutral buffered formalin (NBF) for hematoxylin and eosin (H&E) staining.

### 5.6 Subacute toxicity study (repeated dose)

A subacute toxicity study was performed according to the protocol described in OECD 407 for oral toxicity study in rodents (repeated dose for 28 days). For the subacute study, the rats were divided into five groups (n = 6), and PSPL extract was administered orally at 50 (ST 50), 250 (ST 250), 500 (ST 500), and 1,000 mg/kg (ST 1000), once daily. The subacute control (SC) group received normal saline for 28 days. The subacute satellite control group (SSC) received normal saline, while the subacute satellite group (SST 1000) was orally administered 1,000 mg/kg of PSPL extract for 28 days. Both the SSC and SST 1,000 mg/kg fed groups were observed for any behavioral abnormalities and treatment-related effects for an additional 14 days without any treatment. All rats were under constant observation for the whole treatment duration. The rats were observed for any abnormalities until euthanasia. All rats were euthanized with CO_2_ overdose, and their blood was collected in an EDTA tube for biochemical analysis. Organs such as the liver, kidney, pancreas, retina, aorta, and heart were harvested, weighed, and kept in 10% NBF for H&E staining.

### 5.7 Full blood count and plasma biochemistry

Blood collected in the EDTA tube was subjected to centrifugation at 25,000 rpm for 15 min to obtain serum. Automated BC-2800 VET/Mindray machine was used to analyze plasma biochemistry levels such as red blood cell count (RBC), hemoglobin concentration (Hb), hematocrit (HCt), mean corpuscular volume (MCV), mean corpuscular hemoglobin (MCH), mean corpuscular hemoglobin concentration (MCHC), platelets (Pt), and white blood cell count (WBC). Alere Cholestech LDX^®^ Analyzer (Alere, United Kingdom) was used to measure the liver profile (aspartate transaminase [AST], alanine transaminase [ALT], alkaline phosphatase [ALP], total proteins, albumin, gamma-glutamyl transferase [GGT], and total and direct bilirubin) and renal profile (ions including sodium [Na], potassium [K], and chloride [CI^−]^, urea, and creatinine).

### 5.8 Histopathological analysis

All organs (liver, kidney, pancreas, retina, aorta, and heart) fixed in 10% of NBF were sectioned to obtain paraffin ribbons, followed by fishing of the ribbon from the water bath (40°C–45°C) and fixation to a glass slide. The slides were then stained with H&E as described elsewhere and observed by microscopy (Balan et al., 2021). All images were captured and verified by a certified pathologist from UPM.

### 5.9 Statistical analysis

Statistical analysis was performed using IBM SPSS Statistics for Windows, version 26.0, and all results were expressed as means ± standard error of the mean (SEM) for body weight, food consumption, and calorie intake, while normality tests were run for all data. One-way ANOVA and *post hoc* Tukey tests were used to analyze the significant difference among groups. *P* < 0.05 was defined as a statistically significant result.

## Data Availability

The original contributions presented in the study are included in the article/Supplementary Material. Further inquiries can be directed to the corresponding authors.
